# Long‐term demographic decline and late glacial divergence in a Californian paleoendemic: *Sequoiadendron giganteum* (giant sequoia)

**DOI:** 10.1002/ece3.2122

**Published:** 2016-04-12

**Authors:** Richard S. Dodd, Rainbow DeSilva

**Affiliations:** ^1^ Department of Environmental Science Policy and ManagementUniversity of CaliforniaBerkeleyCalifornia94720

**Keywords:** Demographic change, genetic structure, giant sequoia, Pleistocene, population decline, Sierra Nevada

## Abstract

Mediterranean ecosystems comprise a high proportion of endemic taxa whose response to climate change will depend on their evolutionary origins. In the California flora, relatively little attention has been given to the evolutionary history of paleoendemics from a molecular perspective, yet they number among some of the world's most iconic plant species. Here, we address questions of demographic change in *Sequoiadendron giganteum* (giant sequoia) that is restricted to a narrow belt of groves in the Sierra Nevada Mountains. We ask whether the current distribution is a result of northward colonization since the last glacial maximum (LGM), restriction of a broader range in the recent past (LGM) or independent colonizations in the deeper past. Genetic diversity at eleven microsatellite loci decreased with increasing latitude, but partial regressions suggested this was a function of smaller population sizes in the north. Disjunct populations north of the Kings River were divergent from those south of the Kings River that formed a single cluster in Bayesian assignment tests. Demographic inferences supported a demographic contraction just prior to the LGM as the most likely scenario for the current disjunct range of the species. This contraction appeared to be superimposed upon a long‐term decline in giant sequoia over the last 2 million years, associated with increasing aridity due to the Mediterranean climate. Overall, low genetic diversity, together with competition in an environment to which giant sequoia is likely already poorly adapted, will pose major constraints on its success in the face of increasing aridity.

## Introduction

The relationship between genetic diversity and species' range is a fundamental question in conservation genetics. Although meta‐analyses have suggested that restricted species on average have less genetic diversity than their widespread congeners (Karron 1987; Hamrick and Godt 1990; Cole 2003), many single‐species studies contradict this rule (Delgado et al. 1999; González‐Astorga and Castillo‐Campos 2004; Eliades et al. 2011; Rosas Escobar et al. 2011). Clearly, the origins of restriction of species' ranges play a major role in determining the levels of genetic diversity and population divergence in extant populations. The subsequent evolution of population structure and genetic diversity lie at the heart of conservation biology as they identify divergent lineages that may comprise different adaptive norms and evolutionary potential. For example, species may be locally restricted to their place of origin from which they have not yet had the time or the ability to disperse, or they may be the relicts of once more widespread ranges (Stebbins and Major 1965). In yet other cases, the contemporary distribution of paleoendemics may have arisen from dispersals that have tracked past climate changes. Expectations of the partition of genetic diversity are very different under these contrasting evolutionary scenarios; the former should show low population divergence and low population diversity as the new lineage begins exploiting habitat space, whereas relictual populations might be genetically divergent because of the effects of population isolation and genetic drift. For paleoendemics that have dispersed, the invasion history can result in complex patterns of genetic structure depending on whether contemporary populations originated from single or multiple migration events. In reality, these are the extremes of an eco‐evolutionary gradient that depends on the degree of long‐term stability of populations and the time‐scale over which new populations have expanded, or older populations have declined. Simply, from the perspective of long‐term survival of populations faced with habitat fragmentation and climate change, small populations that have remained demographically stable over long periods of time may not require the extreme conservation measures needed for populations that have undergone severe bottlenecks (Peter et al. 2010), so detecting past demographic changes has great importance in directing conservation strategies.

Mediterranean ecosystems provide unsurpassed opportunities for studying the effects of contrasting evolutionary origins on contemporary genetic structure and, ultimately, the potential for survival under future climate change. These ecosystems that are of relatively recent origin (Axelrod 1972; Suc 1984) host exceptionally high numbers of endemic taxa that include recent radiations, relicts of pre‐Mediterranean climatic phases that have survived *in situ*, and paleoendemics that have dispersed into the new Mediterranean habitat (Ackerly 2009). Environmental and physical barriers have limited recent dispersals, as is the case for the California Floristic Province (CAF), with an oceanic barrier to the west, high montane barrier to the east, and climatic barriers to the north (cool humid climate) and to the south (extreme aridity) (Ackerly 2009). As a result, species of the new Mediterranean ecosystems have evolved in isolation and with evolutionary trajectories that depend on their origins and responses to subsequent climatic fluctuations, particularly during the Pleistocene. For many species, major population demographic changes occurred during the cyclical climatic oscillations of the Pleistocene, culminating with the last full glacial (LGM) event (23,000–18,000 years BP) and subsequent Holocene warming (Hewitt 2004). In montane regions, variable topography is likely to have led to idiosyncratic distributions of refugia, including local persistence and shifts in distributions of populations involving complex patterns of altitudinal and latitudinal extirpations and migrations and demographic expansions and contractions (Stehlik et al. 2001; Eckert et al. 2008; Schoville et al. 2011). Extant montane populations represent descendants from *in situ* refugia, or novel postglacial colonization by diffuse migration from contiguous populations, or by long‐distance dispersal. Understanding the evolution of montane paleoendemics poses major challenges as Holocene demographic changes were superimposed on a prior population structure brought about by long‐term bouts of genetic drift and population shifts, but it is these species that are of major concern in conservation biology. Understanding how long populations have followed independent evolutionary trajectories, the past stability of population sizes and migration among populations that have resulted in contemporary genetic structuring are critical in assessing the long‐term survival of conservation units (Paquette et al. 2007; Sly et al. 2010; Karl et al. 2011; Schoville et al. 2011; Reilly et al. 2012).

**Figure 1:**
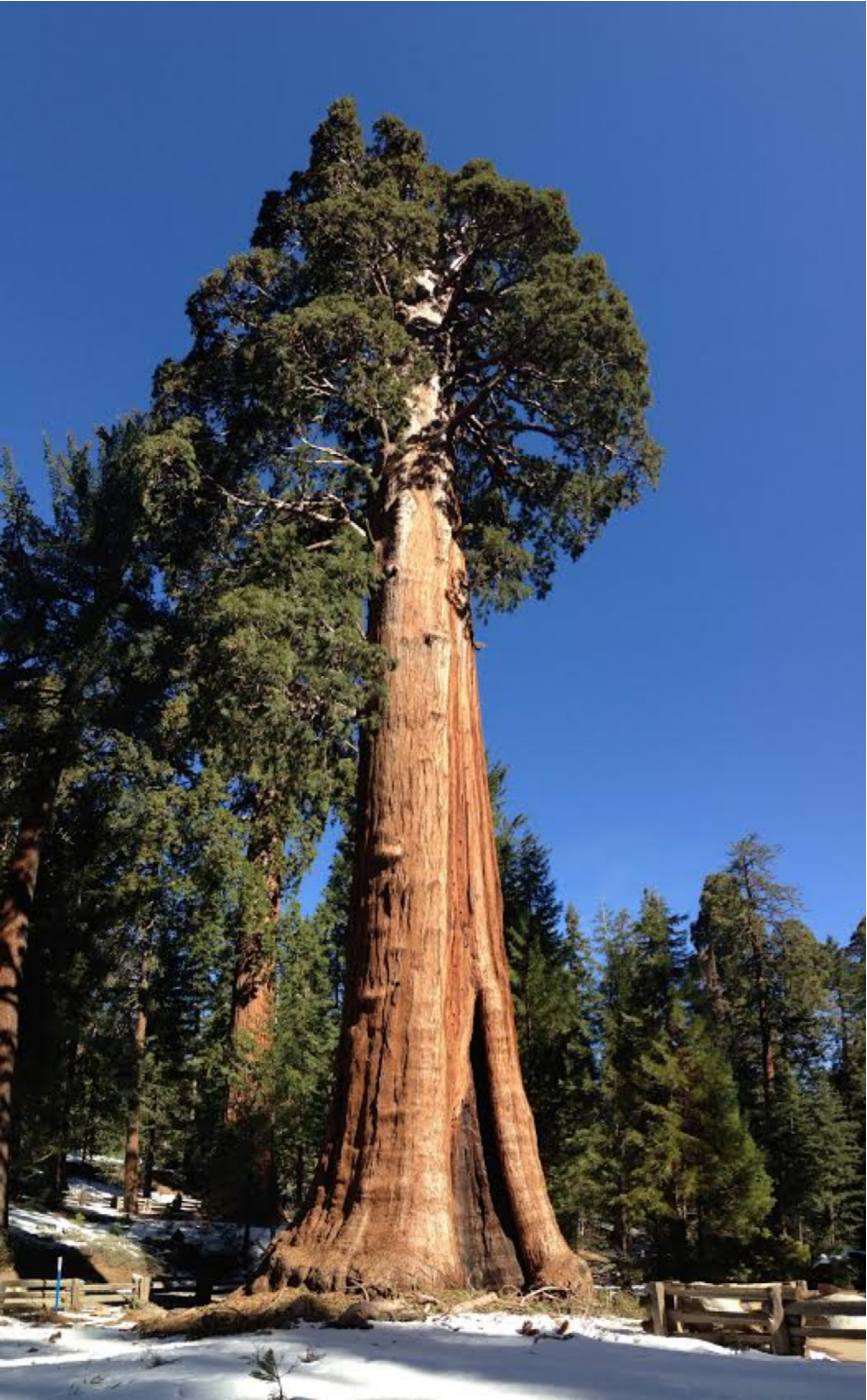
“The Sentinel” giant sequoia in Giant Forest, Kings Canyon National Park.

We focus here on demographic fluctuations and their effect on genetic structure of giant sequoia (*Sequoiadendron giganteum* [Lindl.] Buchholz), a paleoendemic species that occupies the western slope of the Sierra Nevada Mountains of California (Fig. 1). The fossil record suggests that during the Pliocene, populations were present along the eastern flank of the Sierra Nevada, in what is now Nevada (Axelrod 1986). Today, giant sequoia forms a series of groves that are more or less continuous in the southern range, but north of the Kings Canyon, the groves are isolated and well separated. This has led to speculation as to whether isolation of the northern groves is the result of (1) long‐distance seed dispersal; (2) climatic changes that reduced a once more continuous range; or (3) independent origins from ancestral populations on the eastern flank of the Sierra Nevada. We investigate these scenarios using demographic inferences from microsatellite DNA diversity.

## Methods

### Species

**Figure 2:**
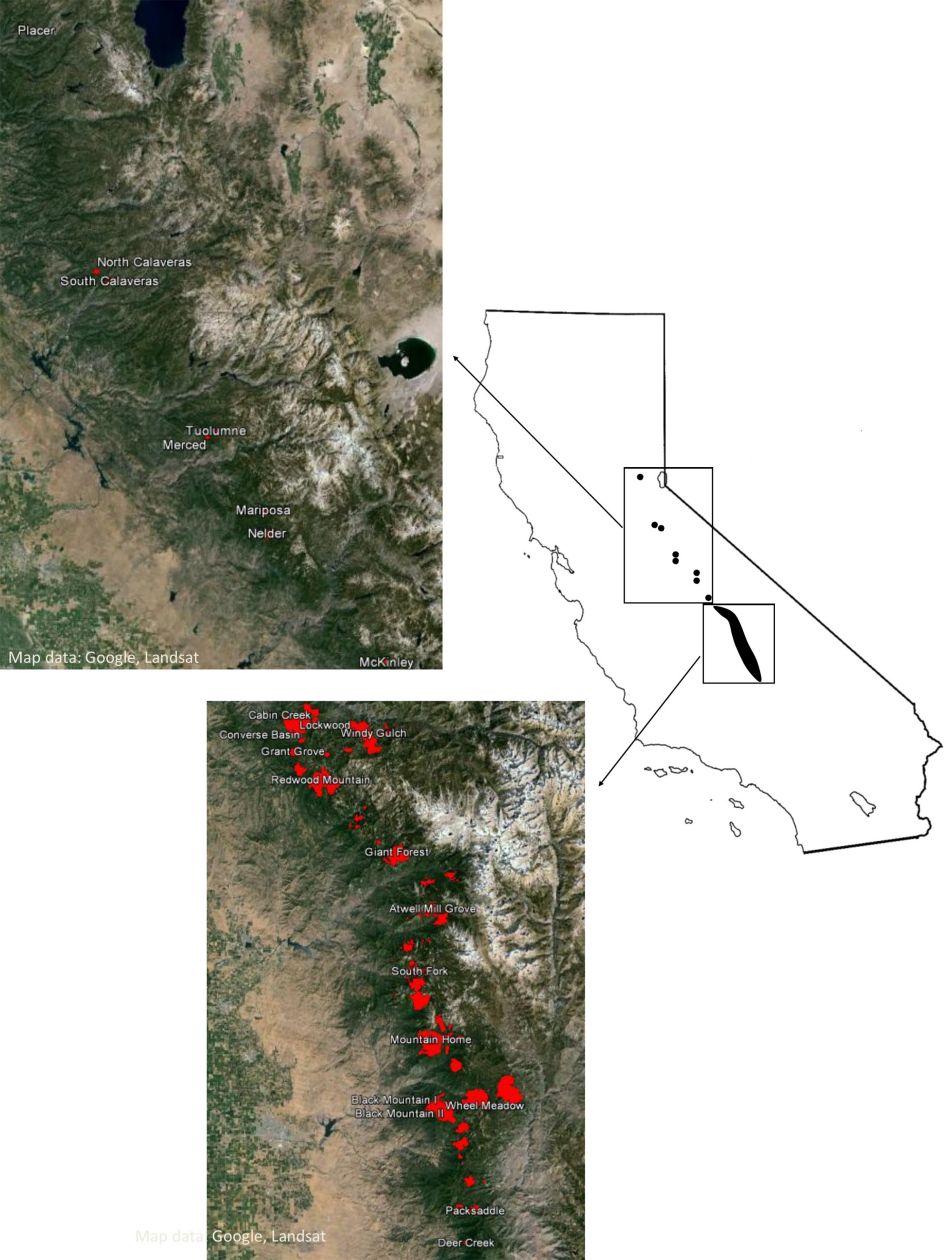
Distribution of giant sequoia groves in the Sierra Nevada Mountains of California. Northern and southern groves shown in separate topographic images. Red shapes denote extant groves, with sampled locations appearing as named groves.

Giant sequoia (*Sequoiadendron giganteum* [Lindl.] Buchholz) is endemic to the western slope of the Sierra Nevada range in Central California. It occurs in about 75 groves stretching in a narrow belt (not more than 24 km wide and about 420 km long) from the American River in Placer County (Placer Grove) to southern Tulare County (Deer Creek Grove). The northern two‐thirds of the range comprise eight small disjunct groves. The remaining groves, including all the large ones, occur from the Kings River drainage south to Deer Creek (Fig. 2). Groves range in size from about 1 ha to 1 619 ha and occupy a total area of about 14 500 ha (Thomas et al. 1980).

### Sampling

Foliage was collected from 357 trees, representing 23 of the natural giant sequoia groves, including 8 northern disjunct groves from Placerville to McKinley and 15 southern groves including Deer Creek, the southernmost limit of the species (Table 1, Fig. 2). The trees were from a clonal orchard at the University of California's Russell Research station that was established in 1981 from seed collections made in 1974 to 1976 [for details see Fins and Libby (1982)]. Briefly, single‐cone collections from different trees (mothers) were made in each of the groves. Seed was germinated, and rooted cuttings were made from young plants grown from the seed. The rooted cuttings were planted out in a design that intended to replicate the seedlings within mothers from each population. Our samples were taken from the rooted cuttings from varying numbers of seedlings per mother tree from each population. We did not include any clonal replicates of seedlings. We recognize that the sampling necessarily includes siblings, but the different mother trees were open‐pollinated and therefore sampled the available pollen pool. To reduce bias in our data analyses, we eliminated samples with an index of relatedness that would suggest more than half‐sib status (see Data analysis below).

**Table 1:** Sample locations and mean genetic parameters for giant sequoia

Grove	Lat	Long	Grove area (ha)	*F*	*N*	*A*_R_	*H*_E_	*H*_O_
Placer	39.057	−120.574	1	4	8	2.08	0.29	0.25
N Calaveras	38.279	−120.302	24	8	29	3.34	0.58	0.50
S Calaveras	38.247	−120.240	184	8	21	3.57	0.63	0.53
Tuolomne	37.769	−119.807	8	7	8	2.65	0.53	0.32
Merced	37.750	−119.839	8	2	8	3.05	0.55	0.56
Mariposa	37.509	−119.604	101	5	15	3.52	0.654	0.51
Nelder	37.435	−119.590	195	8	29	3.73	0.696	0.58
McKinley	37.023	−119.105	22	7	19	3.31	0.570	0.57
Cabin Creek	36.806	−118.941	40	7	21	3.40	0.619	0.55
Converse Basin	36.809	−118.977	1498	10	21	3.71	0.673	0.63
Lockwood	36.793	−118.841	40	4	10	3.52	0.622	0.55
Windy Gulch	36.766	−118.811	405	3	13	4.07	0.683	0.61
Grant	36.750	−118.984	130	2	2	–a	–a	0.55
Redwood Mtn	36.694	−118.916	1271	7	17	3.48	0.593	0.52
Giant Forest	36.565	−118.752	855	5	17	3.72	0.637	0.57
Atwell Mill	36.468	−118.674	383	7	18	3.70	0.631	0.60
Mountain Home	36.358	−118.706	97	4	8	3.70	0.624	0.57
Black Mtn 1	36.230	−118.681	1620	4	17	3.68	0.623	0.54
Black Mtn 2	36.140	−118.513	498	6	30	3.63	0.622	0.49
Wheel Meadow	36.118	−118.679	669	9	13	3.83	0.650	0.60
Packsaddle	36.102	−118.649	669	5	16	2.94	0.555	0.47
South Fork	35.929	−118.592	137	5	12	4.00	0.653	0.64
Deer Creek	35.872	−118.609	21	4	5	2.82	0.520	0.33

Numbers of families sampled per grove (*F*), number of individuals sampled per grove (*N*), allelic diversity (*A*_R_) adjusted to sample size 5, expected heterozygosity (*H*_E_), observed heterozygosity (*H*_O_).

a Sample size too small for estimate.

### Data collection

The DNA was extracted using the cetyltrimethyl ammonium bromide (CTAB) method, following Cullings (1992). Individuals were genotyped at eleven microsatellite loci, including ten dinucleotide repeats (GS_40527, GS_29596, GS_31267, GS_34305, GS_17786, GS_36493, GS_31670, GS_39473, GS_30133, and GS_33118) and one trinucleotide repeat (GS_7365) previously designed for *S. giganteum* by DeSilva and Dodd (2014). All DNA amplifications were carried out using an 18‐bp M13 tail attached to the forward primers and a universal fluorescent‐labeled M13 primer, employing the method described by Schuelke (2000). PCRs consisted of a 10 *μ*L solution containing 200 *μ*mol/L of each dNTP (Roche), 0.5 units of TAQ DNA polymerase (Qiagen, Redwood City, CA), 1 *μ*L −10x PCR buffer with 15 mmol/L MgCl_2_, 0.4 *μ*mol/L reverse, 0.16 *μ*mol/L FAM‐labeled M13 primer, and approximately 20 ng of extracted DNA. The solution was run through an initial denaturation at 95°C for 15 min, followed by 30 cycles of 30 sec at 95°C, 45 sec at 56°C, and 45 sec at 72°C. This was followed by 8 cycles of 95°C for 30 sec, 53°C for 45 sec, and 72°C for 45 sec. Final extension consisted of 30 min at 72 C. Then, 1.5 *μ*L of the PCR product was mixed with a solution of 8 *μ*L formamide and 0.5 *μ*L Genescan LIZ500 size standard (Applied Biosystems, Waltham, MA). The resulting solution was run through an ABI 3730 automated sequencer. Microsatellite fragments were analyzed with Genescan 3.7 and Genotyper 3.7 software (Applied Biosystems).

### Relatedness among samples

We estimated pairwise relatedness coefficients (Lynch and Ritland 1999) among all individuals in GenAlex v. 6.502 (Peakall and Smouse 2006, 2012), see Fig. S1 for the frequency distribution of coefficients. Individuals were ranked according to the number of times they appeared in pairwise comparisons with coefficients greater than 0.25, expected for half‐sibs (a single sample could be related to a number of other samples). An iterative procedure was used to sequentially remove the individual with the highest rank; after removal of an individual, rankings were recalculated before removing the next individual. This procedure was followed until no relatedness coefficients greater than 0.25 remained. An exception was made for the Placer population, for which all samples had pairwise coefficients within the population greater than 0.25. This procedure resulted in reducing the number of samples from 357 to 319.

### Genetic diversity

Microsatellite loci were tested for deviation from Hardy–Weinberg expectations using unbiased Markov chain exact tests in GENEPOP v. 3.4 (Raymond and Rousset 1995; Rousset 2008), with 10 000 dememorizations, 1000 batches, and 10 000 iterations per batch. Linkage disequilibrium among loci for each population was tested in LinkDos (Garnier‐Gere and Dillman 1992). Sequential Bonferroni corrections were made to probabilities for multiple tests according to Rice (1989). Quality of microsatellite data was further tested for scoring errors and null alleles using MICRO‐CHECKER (Van Oosterhout et al. 2004). FSTAT v.2.9.3 (Goudet 1995) was used to compute gene diversity (*H*_E_) and allelic richness (*A*_R_) (adjusted to the smallest population size of 5 individuals at Deer Creek (the two individuals from Grant were removed for this test)).

### Isolation by distance

Because of the more or less linear distribution of giant sequoia in the Sierra Nevada, we recognize that a pattern of isolation by distance (IBD) may confound discrete population genetic structure. Pritchard et al. (2000) caution that where IBD is significant, interpretation of STRUCTURE results will be challenging. We tested for a signal of IBD using IBDWS Vers. 3.23 (Jensen et al. 2005). To avoid the risk of bias in estimating population similarity, we removed all populations with less than 10 samples (Placer *n* = 8, Merced *n* = 8, Tuolumne *n* = 8, Deer Creek *n* = 5). Because geographic distance could be confounded by the strong geographic pattern of northern disjunct populations and the southern more continuous range, we created a binary indicator matrix, in which pairwise population comparisons were coded as 0 if both populations in the comparison came from a like group and 1 if they came from a unlike group. Four groups were formed on the basis of their approximate contiguity: (1) two Calaveras groves, (2) Mariposa and Nelder groves, (3) McKinley grove, and (4) remaining groves south of the King River. Tests of IBD were performed with 30,000 randomizations of Rousset's distance [*F*_ST_/(1 − *F*_ST_)] and geographic distances based on population centroids of latitude and longitude coordinates. Rousset (1997) recommends plotting genetic distance against the log of geographic distance for two‐dimensional habitats and against geographic distance in one‐dimensional habitats. However, he qualifies this by saying that it is not obvious when an elongated habitat should be treated as one or two dimensional. Populations in the northern range of giant sequoia may best fit a one‐dimensional model, but in the south, a two‐dimensional model is more appropriate. We ran Mantel tests using both the log of geographic distance and raw distances, but because the tests yielded comparable results, we report only data from the log of geographic distance here.

### Population structure

We used two Bayesian model‐based approaches to identify population genetic structure: a spatially explicit model in BAPS v 6.0 (Corander et al. 2008) and an equilibrium‐based, Markov Chain Monte Carlo model performed in STRUCTURE v.2.3.4 (Pritchard et al. 2000) with no spatial priors. Both BAPS and STRUCTURE are intended to identify discrete variations in allele frequencies that likely arise from the effects of genetic drift associated with the past demographic events. Whereas STRUCTURE makes use of Markov chain Monte Carlo simulations and can only use population of origin identity as a prior for informing the analysis, BAPS makes use of analytical and stochastic methods that incorporate spatial coordinates. We applied the two approaches in this study, because on the one hand, incorporating spatial information is an efficient approach to detecting weak structure in genetic data, but, on the other hand, detracts from one of the objectives of detecting structure from blind data.

Because we did not have the original coordinates for each sampled individual, we randomly selected their latitude and longitude coordinates from Google Earth maps of each grove. For the purposes of this study, we were not interested in within‐grove fine‐scale spatial structure, so the random coordinates were intended only to provide unique coordinates for each sample. We inferred the optimal partition in BAPS using the spatial clustering of groups' option. In the initial runs, we set *K* (the number of groups) to vary from 2 to 23, to obtain an estimate of the number of partitions. For final runs, we set *K* to vary from 2 to 15, and we ran 10 simulations at each value of *K*. Subsequently, we ran fixed *K* group partition comparisons to test for the most likely evolutionary‐based grouping of sampled populations. We tested four scenarios: (1) two groups: northern disjunct stands (including McKinley grove and groves further north) and the remaining southern groves; (2) six groups: the same north–south divide as in (1), but northern groves were separated into spatially distinct groups (Placer, N Calaveras + S Calaveras, Tuolumne + Merced, Mariposa + Nelder, and McKinley); (3) nine groups as in (2), but all northern groves were treated as separate; and (4) nine groups, divided by major drainage systems (American River, Tuolumne River, North Fork of Kings River, main branch of the Kings River, Marble Fork of Kaweah River, and Middle Fork of the Kaweah River). We set uniform priors of 0.25 to each of the scenarios.

For STRUCTURE, we inferred the number of genetic clusters (*K*) present in the dataset and assigned individuals to these clusters, using the admixture model with correlated allele frequencies, with no a priori information about collection sites. For all final simulations performed in STRUCTURE, we used a “burn‐in” period of 5 × 10^4^ followed by a run length of 5 × 10^5^ MCMC iterations, which were sufficient to give a stable *α* and estimate of the log probability of the data. For preliminary runs, we included the whole dataset with *K* ranging from 1 to 23 (the number of sampled populations). These preliminary runs indicated minor changes in log probability of the data for *K* greater than 8; therefore, for final MCMC runs, we selected the upper *K* value as 8 and ran 10 simulations for each value of *K*. To estimate the most likely *K* value, we used the ΔK statistic (Evanno et al. 2005) by inputting STRUCTURE output into STRUCTURE HARVESTER (Dent and vonHoldt 2012). Results are displayed using the program DISTRUCT (Rosenberg 2004).

### Testing evolutionary scenarios

To elucidate the evolution of population structure, we first tested for a signal of ancient divergence among populations. This is possible by comparing the observed *R*_ST_ with a value obtained after permuting allele sizes within loci (*R*_STperm_). Permuting in this way erases the effect of the stepwise mutational model of microsatellite alleles, but retains the divergence among populations due to allele frequency differences and approximates *F*_ST_ (Hardy et al. 2003). We performed analyses in SpageDi V 1.5 (Hardy and Vekemans 2002), with 20 000 permutations for the allele permutation tests.

**Figure 3:**
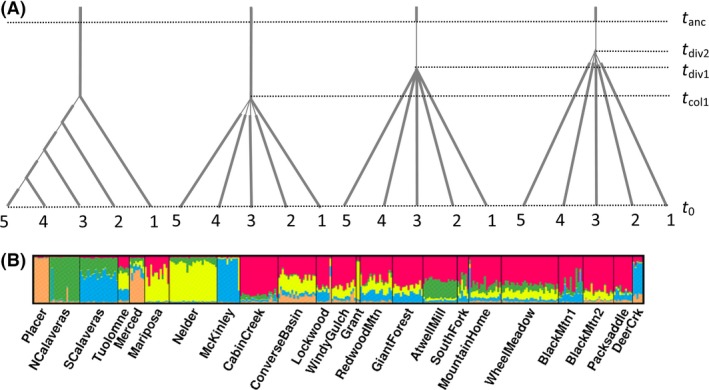
(A) Evolutionary scenarios used in DIYABC. From left to right: 1. sequential colonization northward; 2. mass colonization of northern groves from southern groves; 3. divergence following range reduction during the Pleistocene; and 4. ancient independent colonizations from the eastern Sierra Nevada. All colonization events were followed by a bottleneck shown as thin line. *t*_col1_: time of first colonization northward for the sequential scenario and for all northern colonizations for the mass colonization model; *t*_div1_: divergence time for scenario 3 Pleistocene divergence; *t*_div2_: divergence time for scenario 4 ancient colonization; *t*_anc_: time of ancestral change in population size. (B) Output from STRUCTURE (Pritchard et al. 2000) showing individual assignments to clusters. Figure produced using DISTRUCT (Rosenberg 2004).

To infer the past demographic processes, we performed tests of fit of parameters derived from the simulated data to those estimated from the observed data, within an approximate Bayesian computation (ABC) framework using DIYABC v. 2.0 (Cornuet et al. 2008, 2014). DIYABC is computationally efficient and allows inclusion of many competing evolutionary scenarios with varying demographic complexity. Based on results from population structure described later under Results, we ran our simulations on the assumption that the present‐day groves south of the Kings River drainage (south of McKinley) constituted a single cluster and could therefore be treated as one single population. The McKinley grove and groves further north were treated as being divergent to some degree. However, because of the very close proximity of some of the northern groves, these were pooled for further analyses: North Calaveras and South Calaveras, Tuolumne and Merced, and Mariposa and Nelder. The Placer grove was omitted because of its extremely small size and low genetic diversity. The present‐day range of giant sequoia has drawn speculation as to the timing and origin of stands on the western slope of the Sierra Nevada. It has been questioned whether northern populations are the result of long‐distance dispersal, of contraction of a broader range, or the result of independent dispersals from the eastern flank. Therefore, we evaluated three broad competing scenarios, as shown in Figure 3:

(1) Colonization of northern groves from the south during the late Pleistocene (recent colonization). Here, it was assumed that migration northward was the most likely direction, given the larger populations and greater genetic diversity (see Results) in the south. Within this scenario, we tested two modes of colonization as follows; stepping stone colonization and mass colonization. (2) restriction of a former more continuous range on the western slope and (3) separate migrations from a distribution on the eastern slope of the Sierra Nevada as follows:

Scenario 1a: *Northward stepping‐stone colonization model*: McKinley colonized from the southern group and provided seed for colonization of the Nelder and Mariposa groves. In turn, Nelder and Mariposa provided seed for colonization of Merced and Tuolumne groves. Merced and Tuolumne provided seed for the Calaveras groves. Under these scenarios, it was assumed that the founder populations were small, and so we simulated initial bottlenecks.

Scenario 1b: *Northward mass colonization model*: All northern groves were colonized at the same time from the southern group. Again, the founder populations were assumed to be small and were simulated with bottlenecks.

Scenario 2: Divergence among northern groves and the southern group of groves following restriction in size of an ancestral population during the late Pleistocene. This scenario would suggest a broader and more continuous range prior to the last glacial.

Scenario 3: Independent origins from an ancestral population in the deep past. This scenario would be consistent with early Pleistocene migrations across the Sierra Nevada from populations occupying the eastern slope.

We set uniform priors for all demographic parameters that were assigned minimum and maximum values and conditions (see Table S4). The microsatellite loci were divided into two groups: (1) all dinucleotide repeat loci and (2) the single trinucleotide repeat locus. Default values were used for the microsatellite mutation models. We estimated the following summary statistics: mean number of alleles, mean genic diversity, and mean allele size variance for each population and *F*_ST_ and *δμ* ^*2*^ among population pairwise parameters. For each scenario, we ran 4 x 10^6^ simulated datasets to estimate posterior probabilities using logistic regression. We selected the scenario with the highest posterior probability and estimated the values of the associated demographic parameters. To evaluate confidence in the choice of scenario, we created 500 pseudo‐observed datasets (i.e., datasets simulated with known parameters; Cornuet et al. 2010) in order to evaluate type I (proportion of datasets where the selected scenario did not have the highest posterior probability compared with competing scenarios) and type II error rates (proportion of datasets that had the highest posterior probability for the chosen scenario, but simulated with competing scenarios). Model checking was performed by simulating 10,000 datasets using summary statistics that were not included in the training set for model discrimination (mean Garza Williamson's M, and the two sample statistics mean number of alleles, mean size variance, and shared allele distance). To assess precision of parameter estimation, we computed the relative median of the absolute error (RMAE) on 500 pseudo‐observed datasets simulated under the best‐fit scenario (Cornuet et al. 2010).

Splitting times were converted to absolute scales assuming a range of generation times for this long‐lived woody plant. Generation time in giant sequoia was estimated following Lande (1982):

T=α+s/λ−s,whereλ≈1

We set time to sexual maturity (*α*) as ranging from 60 to 150 years (Weatherspoon 1990) (although earlier cone production has been reported, significant and regular cone production is delayed in giant sequoia) and s, the life curve ≈ 0.995, so that generation time in giant sequoia was estimated to range from ~ 260 to 350 years. Here, we assumed an average of 305 years.

## Results

### Genetic diversity

Hardy–Weinberg equilibrium (HWE) was rejected for only eight of the 231 population–primer combinations (see Table S1). Of the eight combinations not in HWE, five were for primers 4 and 6 in the northern disjunct group of populations. We considered the number of rejections of HWE to be low and not to justify the removal of populations or primers in further analyses. Among all populations, we detected 45 primer pair combinations not in linkage equilibrium, out of a total of 1232 tests (see Table S2). These were scattered across populations and primer combinations suggesting that the instances of LD would not seriously affect further analyses. No evidence for null alleles, or scoring errors, was detected.

We detected a total of 118 alleles across the 11 loci (mean of 10.7 alleles per locus). Across populations, genetic diversity measured as allelic richness (*A*_R_) ranged from 2.08 at Placer to 4.07 at Windy Gulch and measured as expected heterozygosity (*H*_E_) from 0.29 at Placer to 0.70 at Nelder (Tables 1 and S1). We explored patterns of genetic diversity in relation to the following: (1) latitude (L) that might result if groves were the result of a wave front of colonization, (2) grove size assuming a drift effect in smaller populations (*N*), and (3) sample size that might bias diversity estimates (*n*). Partial correlations showed no significant effect of latitude on allelic diversity (*r* _ar__L.*Nn* _= −0.178, *P* = 0.455) after accounting for the effects of grove size (*N*) and sample size (*n*), but significant effects of grove size (*r* _ar__L*N*.L*n* _= 0.560, *P* = 0.010) and sample size (*r* _ar__n.L*N* _= 0.548, *P* = 0.012). No significant partial correlations were found for expected heterozygosity (*r*_HEL.*Nn* _= −0.374, *P* = 0.104; *r*_HEL.*Nn* _= 0.274, *P* = 0.242; *r*_HEL.*Nn* _= 0.31, *P* = 0.184).

### Isolation by distance

Rousset's distance was significantly correlated with the log of geographic distance (*r* = −0.456, *P r* ≥ 0 = 0.001) and with the indicator matrix (*r* = −0.563, *P r* ≤ 0 = 0.001). However, after controlling for the indicator matrix, the partial correlation of genetic similarity and geographic distance was not significant (*r* = 0.108, *P r* ≤ 0 = 0.183), whereas genetic distance was significantly correlated with the indicator matrix after controlling for geographic distance (*r* = 0.383, *P r* ≤ 0 = 0.04). Because of the lack of a significant signal of IBD, we continued with tests to detect population genetic structure.

### Structure of diversity

An optimal partition of 11 clusters was inferred by BAPS spatial clustering of individuals (Table S3). Of these clusters, two comprised only two individuals each, so that the bulk of individuals was assigned to one of 9 clusters. Individuals from each of the northern groves were either exclusively (Placer, South Calaveras, Merced) or mostly assigned (North Calaveras, Mariposa, Tuolumne, Nelder, and McKinley) to single clusters. All southern groves were almost exclusively assigned to a single cluster. Among the four partition comparisons, posterior probabilities were estimated as Scenario 1 Prob 0, Scenario 2 Prob 0, Scenario 3 Prob 1, and Scenario 4 Prob 0, indicating that the genetic data best fit the third scenario of nine groups including single‐grove clusters for all northern groves and a single cluster for groves south of McKinley.

The log probability of the data for increasing values of *K* in STRUCTURE increased more slowly from *K* = 6–8 (Fig. S1). The Δ*K* parameter [Evanno et al. 2005; ] was maximum for *K* = 5 (Fig. S2). Assignments were mostly to one of four single clusters within northern populations, with the exception of Tuolumne and Merced groves, whereas in the south, there was no clear correspondence between populations of origin and cluster assignment (Fig. 3). A break in structure assignments appeared between the McKinley and Cabin Creek groves, corresponding with the distributional break in the Kings River watershed. At the individual level, admixture (shared assignments to clusters) was relatively low in the north, but became more important within individuals in the south.

### Evolutionary inferences

Global *R*_ST,_ with northern groves treated as the separate groups and the southern groves as a single group, was significantly greater than permuted *R*_ST_ (*R*_STobs_ = 0.120; *R*_STperm_ = 0.088; *P R*_STobs* *_> *R*_STperm_ = 0.042). The permuted *R*_ST_ is equivalent to an *F*_ST_ estimate, so that a significantly observed *R*_ST_ indicates a phylogeographic signal as a result of mutational divergence. Also, we tested for phylogeographic signal for northern and southern populations separately. Observed *R*_ST_ was greater than permuted *R*_ST_ for the northern populations, but the difference was not significant (*R*_STobs_ = 0.172; *R*_STperm_ = 0.143; *P R*_STobs _> *R*_STperm_ = 0.22). For southern populations, permuted *R*_ST_ was slightly greater than observed *R*_ST_ (*R*_STobs_ = 0.066; *R*_STperm_ = 0.076; *P R*_STobs_ > *R*_STperm_ = 0.68).

Among evolutionary models tested in DIYABC, simultaneous divergence among northern groves and the southern group of groves following restriction in size during the late Pleistocene (scenario 2) was the most likely scenario (Table 2). The 95% confidence limits around the posterior probability of 0.904 did not overlap with confidence limits for any of the other scenarios. Type 1 and Type 2 error rates for scenario 2 were 0.03 and 0.07, respectively. As expected, current effective population sizes were small for northern groves relative to the combined southern group (Table 2). The sum of current effective population sizes for the 5 groups was approximately four times greater than the effective population size of the combined population at divergence (*N*_div _= 1870; 95% CI; 589–6640), but about one‐third of the ancestral population size of 23,000 (95% CI; 2380–92,900). RMAEs for parameter estimates of contemporary populations were low, with higher values for the past demographic estimates: *N*_anc _= 0.37 and *t*_anc _= 0.35 (Table 2). As is common with such demographic inferences, confidence intervals were large; however, the median values of timing of events are consistent with the past climatic changes. The most likely scenario among those tested suggests a reduction in population size that led to a more or less simultaneous divergence among the 5 groups about 21 kya (95% CI; 6.7–53.1). An overall decline in giant sequoia is likely to have occurred about 2.3 Mya (95% CI; 0.6–3.0), corresponding with the onset of the Pleistocene.

**Table 2:** Posterior parameter estimates from DIYABC computations assuming a mean generation time of 305 years

Parameter	Median	95% CI	RMAE
N1	5380	1460–9720	0.223
N2	480	176–684	0.208
N3	284	120–938	0.198
N4	592	327–694	0.189
N5	329	91–664	0.194
*N*_div_	1870	589–6640	0.281
*N*_anc_	23,000	2380–92,900	0.345
*t*_d_ generations (kya)	68.3 (20.8)	22–174 (6.7–53.1)	0.219
*t*_anc_ generations (kya)	7610 (2321)	1840–9910 (561–3023)	0.370

N1: effective population size of southern groves; N2: effective population size of McKinley grove; N3: effective population size of Mariposa and Nelder groves; N4: effective population size of Tuolumne and Merced groves; N5: effective population size of North and South Calaveras groves; *N*_div_: effective size of population prior to divergence; *N*_anc_: effective population size of ancestral population prior to decline; *t*_d_: time since divergence; *t*_anc_: time since decline.

## Discussion

The redwoods of California are classic examples of paleoendemic taxa whose origins predate the Mediterranean ecosystem that they currently occupy. Giant sequoia is renowned for its restriction to a series of groves at mid‐elevations of the western slope of the Sierra Nevada that is more or less continuous in the southern third of the range, but strongly disjunct in the north. This distribution has led to questions concerning the origins of giant sequoia in the Sierra Nevada and the processes that have led to its current distribution. We provide evidence that climatic events during the Pleistocene had important consequences for the current levels and distribution of genetic diversity. A picture emerges of a species that has declined in numbers over the last 2 My, with a distribution that was most recently fragmented at the onset of the last glacial maximum.

### Origin of low genetic diversity

Early isozyme studies revealed relatively high genetic diversity in coniferous tree species attributed to widespread pollen dispersal and predominantly outcrossing breeding systems and low genetic differentiation among populations (Hamrick and Godt 1990, 1996). More recently, studies of hypervariable microsatellite loci have provided further support for this trend. However, microsatellite diversity (average number of alleles per locus = 10.7 for the total sample, average allelic diversity per population = 3.4) in giant sequoia is low compared with other species. For example, 25 alleles per locus were reported for the widespread taxon *Pinus ponderosa* (Potter et al. 2015), with an average allelic richness per population of 5.5, and 24 alleles per locus were detected in the Japanese paleoendemic *Cryptomeria japonica,* with an average allelic diversity per population of 5.6 (Takahashi et al. 2005). For other members of the Cupressaceae, Pandey and Rajora (2012) reported 16 alleles per locus and average allelic diversity of 6.2 among low‐diversity peripheral populations of *Thuja occidentalis*, and O'Connell et al. (2008) found 23.6 alleles per locus with an average of 10.3 alleles per locus across coastal and interior populations of *Thuja plicata*. Admittedly, comparisons of microsatellite allelic diversity across species should be treated with caution because of the potential bias associated with the microsatellite development. However, our loci were developed from genomic DNA, and over 11 loci should provide a robust measure of microsatellite polymorphism. Interestingly, although allelic diversity was below average, levels of heterozygosity were moderate, suggesting that inbreeding is unlikely to have contributed significantly to the decreased genetic diversity. *H*_E_ was higher than *H*_O_ in 20 of the 23 populations analyzed, suggesting that some inbreeding may occur, but this could be limited to early developmental stages, because the plant material used for these analyses came from clones of seedlings grown in a plantation setting where mortality may be limited. Early inbreeding depression is common in outcrossing species such as many trees (Kremer et al. (2012). Allelic diversity declined northward, but this did not appear to be related to founder effects because the lower allelic diversity was more closely associated with grove size than with latitude and because no significant clinal pattern in observed heterozygosity was detected. Therefore, it seems more likely that the low allelic diversity is due either to bias in microsatellite development or to loss of alleles. We favor the latter because our microsatellites were selected for levels of polymorphism and ease of scoring from a much larger panel of loci (DeSilva and Dodd 2014) and because there were gaps in the range of microsatellite alleles that would be an indicative of random loss of alleles through drift.

Our detection of low genetic diversity confirms an earlier report of low diversity at isozyme loci that were interpreted to be the result of ancient bottlenecking as ancestral populations of giant sequoia dispersed southwestward from the present‐day Idaho (Fins and Libby 1982). However, our approximate Bayesian computations suggest a more recent demographic decline that began about 2.3 Mya, probably coincident with major climatic changes at the end of the tertiary. Giant sequoia appears in the fossil record of western Nevada from the mid‐ and late‐Miocene floras that were characterized by relatively warm, but humid climates (Axelrod 1986), but was lost from eastern Nevada during the late Pliocene as climate became more arid and the full Mediterranean climate became established (Millar 1996). Our data suggest that a long‐term decline in giant sequoia began at this time, probably as a result of relatively poor adaptation to the more xeric summer conditions and possibly in competition with more aggressive mixed‐conifer forest species.

### Population structure

Postglacial long‐distance dispersal has been invoked as a possible explanation for the disjunct distribution of northern groves of giant sequoia. If this was the case, we should expect weak population differentiation. However, our Bayesian clustering analyses provided an evidence of population structure that defines a discontinuity around the Kings Canyon Divide, consistent with the break between the southern and northern disjunct groves and further divergence among the northern groves. The spatial Bayesian clustering performed in BAPS revealed a strong pattern of divergence in which all northern groves formed unique clusters. Bayesian clustering can overestimate population structure if IBD contributes to patterns of genetic diversity (Frantz et al. 2009). However, this was unlikely for giant sequoia, because partial Mantel tests indicated that isolation of the northern populations was the determining factor in the apparent association between geographic distance and genetic similarity. The admixture analysis in STRUCTURE clearly showed low levels of admixture in northern groves (with the exception of the Tuolumne and Merced groves), indicating that recent gene flow was probably lacking. By contrast, southern groves were strongly admixed that would suggest this group of groves is held together by recent or contemporary gene flow. Confirmation of contemporary gene flow awaits results from fine‐scale studies that we are currently conducting.

### Demographic processes

To investigate past demographic changes of giant sequoia, we inferred population size changes and splits from genetic data using approximate Bayesian computations. Climatic cycles during the Quaternary are known to have had important demographic consequences for species, including the distribution of genetic diversity (Hewitt 1996). The paleoecological record for the Sierra Nevada indicates colder and drier conditions during the late glacial, with wetter conditions developing about 12.5–10 kya (Anderson 1990). A warming and drying trend began about 10 kya and continued until about 6.5–5 kya, when climate became cooler and more mesic (Davis and Moratto 1988; Anderson 1990; Edlund and Byrne 1991). The pollen record for giant sequoia over this period indicates the presence in sites distant from the present‐day groves, Mono Lake 11.5 kya (Davis 1999) and Tulare Lake 26 kya (Atwater et al. 1986), as well as sites at lower or higher elevations than current groves during the early‐ to mid‐Holocene (Cole 1983; Davis and Moratto 1988). In some extant groves in the southern Sierra Nevada, giant sequoia pollen is scarce prior to 4.5 kya, suggesting recent colonization (Anderson 1994; Anderson and Smith 1994). The presence of giant sequoia pollen at some sites and the absence from others has been interpreted as evidence for range shifts during the late Quaternary and has led to the conclusion that giant sequoia has come to occupy its current distribution as recently as 4.5 kya (Millar and Woolfenden 1999). Although our demographic inferences suggest population increase since divergence at about 21 kya, preliminary runs of DIYABC (not shown here) showed no evidence of very recent expansions (last 5–8 kya). Therefore, our data are not fully consistent with the pollen record that suggests a recent expansion (Anderson 1994; Anderson and Smith 1994). Furthermore, summing current effective population sizes of the separate populations likely overestimates contemporary total effective population size, so that a postglacial population expansion is likely to be less than the fourfold increase that we have inferred. How can we reconcile past demography based on genetic data with the pollen record that indicates recent expansion? Fluctuations in population size and population spatial shifts have most likely occurred as climate changed since the last glacial maximum. However, montane environments can provide climatic refugia over relatively short distances. Short‐distance shifts in elevation (up or down) and aspect may be sufficient to provide suitable habitat during extreme climate change. The pollen record provides some evidence that this could have been the case for giant sequoia. Davis and Moratto (1988) reported giant sequoia as present at Exchequer Meadow about 11 kya at what would have been the elevational limit for the species. Today, giant sequoia is absent from Exchequer Meadow, but is present just 5 km south at McKinley Grove. Similarly, Cole (1983) reported full glacial pollen of giant sequoia in packrat middens in Kings Canyon National Park. Today, giant sequoia grows 2 km from the midden site, but lack of modern giant sequoia pollen in the middens was interpreted as evidence that at full glacial, giant sequoia would have been no more than 2 km distant. Such examples suggest that range shifts of giant sequoia may have been very local, with extirpations at some locations and colonization at others. If colonization was achieved through slow mass dispersal, this would probably not have greatly influenced the genetic signature of demographic change. Therefore, the genetic data probably accurately reflect general demographic trends, but not local perturbations.

### Future under climate change

Axelrod titled his 1986 manuscript The Sierra redwood (*Sequoiadendron*) forest: End of a dynasty. The demise of giant sequoia was seen as the consequence of fire suppression that encourages competing vegetation and inhibits its germination and establishment. Although today, management of the groves is aimed at providing the conditions suitable for regeneration, the new threat comes from climatic change. It is worrisome that genetic diversity was generally moderate to low and particularly low in small northern groves. Under natural colonization, the northern groves might be expected to serve as source populations for colonization of new suitable habitat at higher latitudes, or at higher elevations, although the latter would likely result in smaller populations with competition among species for limited space. Our inferences of past demographic changes suggest that the decrease in summer rainfall has led to a general decline in giant sequoia that has been exacerbated by cold, dry glacial cycles. If, as predicted, future climate in the Sierra Nevada will entail reduced winter snow pack and reduced summer rainfall, giant sequoia will face increasing pressure to compete in its present range. As suggested by the spatial shifts that likely occurred during the late Pleistocene and Holocene, local displacements may be possible if the rapidity of climate change is slow enough that dispersal and establishment can keep pace. However, if conditions in the current range of the species become drier and warmer, the long‐term decline that we have detected is likely to continue, and the future of this iconic California paleoendemic may be a signal of what can be expected for other Mediterranean ecosystem paleoendemics. In the absence of human intervention to plant in suitable habitat, the dynasty may indeed be at an end.

## Conflict of Interest

None declared.

## Data Accessibility

Raw microsatellite genotype data for the full dataset of 357 individuals will be submitted to datadryad.org if the manuscript is accepted for publication. The individuals removed based on the criterion of 0.25 pairwise relatedness coefficient will be indicated in the microsatellite dataset. Conditions and priors for the DIYABC analyses are in the accompanying Supporting information for online publication.

## Supporting information


**Table S1.** Summary of microsatellite data by population.
**Table S2.** Linkage equilibrium.
**Table S3.** Priors for all demographic parameters used in DIYABC.
**Table S4.** Partition from BAPS spatial clustering of individuals.
**Fig. S1.** Lynch and Ritlands pairwise relatedness coefficients for the full dataset of 357 individuals.
**Fig. S2.** Log probability of the data for increasing values of *K* in STRUCTURE.
**Fig. S3.** Evanno et al. (2005) Δ*K* parameter for increasing values of *K*.
**Fig. S4.** Scenario evaluation in DIYABC.
