# Supplementary material for: Long‐term demographic decline and late glacial divergence in a Californian paleoendemic: Sequoiadendron giganteum (giant sequoia)
Source: Ecol Evol. 2016 Apr 12;6(10):3342–55. doi: 10.1002/ece3.2122 (PMC4870217; doi:10.1002/ece3.2122)
Supplement: Supplementary file 1 — Table S1. Summary of microsatellite data by population. Table S2. Linkage equilibrium. Table S3. Priors for all demographic parameters used in DIYABC. Table S4. Partition from BAPS spatial clustering of individuals. Fig. S1. Lynch and Ritlands pairwise relatedness coefficients for the full dataset of 357 individuals. Fig. S2. Log probability of the data for increasing values of K in STRUCTURE. Fig. S3. Evanno et al. (2005) ΔK parameter for increasing values of K. Fig. S4. Scenario evaluation in DIYABC. [file ECE3-6-3342-s001.docx]

**Supporting Information**

MS Title: Long-Term Demographic Decline and Late Glacial Divergence in a Californian Paleoendemic: *Sequoiadendron giganteum* (Giant Sequoia)

Authors: Richard S. Dodd, Rainbow DeSilva

**Contents**

Table S1. Summary of microsatellite data by population

Table S2. Linkage equilibrium

Table S3. Priors for all demographic parameters used in DIYABC

Table S4. Partition from BAPS spatial clustering of individuals

Fig S1. Frequency distribution of pairwise relatedness coefficients

Fig S2. Log probability of the data for increasing values of K in STRUCTURE

Fig S3. Evanno *et al.* (2005) ΔK parameter for increasing values of K

Fig S4. Scenario evaluation in DIYABC

Table S1. Summary of microsatellite data by population

|  |  |  | 4057 | 7365 | 29596 | 31267 | 34305 | 177867 | 36493 | 31670 | 39473 | 30133 | 33118 |
| --- | --- | --- | --- | --- | --- | --- | --- | --- | --- | --- | --- | --- | --- |
| **Placer** |  | **Alleles** | 1 | 3 | 1 | 6 | 2 | 1 | 1 | 5 | 3 | 2 | 1 |
|  |  | **A_R_** | 1 | 2.25 | 1 | 4.831 | 1.999 | 1 | 1 | 4.1239 | 2.625 | 1.964 | 1 |
|  |  | **H_E_** | 0 | 0.241 | 0 | 0.800 | 0.458 | 0 | 0 | 0.783 | 0.591 | 0.325 | 0 |
|  |  | **H_O_** | 0 | 0.250 | 0 | 0.250 | 0.375 | 0 | 0 | 0.750 | 0.750 | 0.375 | 0 |
|  |  | **HWE** | - | 1.000 | - | **0.000** | 1.000 | - | - | 0.009 | 0.478 | 1.000 | - |
| **N Calaveras** |  | **Alleles** | 4 | 3 | 4 | 5 | 4 | 4 | 4 | 8 | 9 | 4 | 2 |
|  |  | **A_R_** | 3.173 | 2.484 | 3.421 | 3.936 | 2.887 | 3.771 | 3.099 | 3.857 | 5.808 | 2.951 | 1.313 |
|  |  | **H_E_** | 0.567 | 0.446 | 0.669 | 0.750 | 0.605 | 0.748 | 0.567 | 0.620 | 0.859 | 0.528 | 0.062 |
|  |  | **H_O_** | 0.500 | 0.438 | 0.563 | 0.688 | 0.688 | 0.438 | 0.500 | 0.438 | 0.813 | 0.375 | 0.062 |
|  |  | **HWE** | 0.420 | 1.000 | 0.170 | 0.798 | 0.890 | 0.020 | 0.273 | 0.030 | 0.136 | 0.085 | 1.000 |
| **S Calaveras** |  | **Alleles** | 4 | 3 | 8 | 10 | 4 | 3 | 3 | 6 | 12 | 4 | 2 |
|  |  | **A_R_** | 3.404 | 2.966 | 4.126 | 5.331 | 3.647 | 2.676 | 2.215 | 3.776 | 6.045 | 3.660 | 1.442 |
|  |  | **H_E_** | 0.637 | 0.678 | 0.710 | 0.815 | 0.735 | 0.537 | 0.422 | 0.705 | 0.863 | 0.699 | 0.100 |
|  |  | **H_O_** | 0.650 | 0.700 | 0.650 | 0.684 | 0.850 | 0.100 | 0.150 | 0.450 | 0.650 | 0.800 | 0.100 |
|  |  | **HWE** | 0.582 | 0.759 | 0.456 | 0.037 | 0.765 | **0.000** | 0.003 | 0.012 | 0.008 | 0.761 | 1.000 |
| **Tuolomne** |  | **Alleles** | 2 | 3 | 2 | 3 | 3 | 3 | 2 | 5 | 3 | 4 | 2 |
|  |  | **A_R_** | 2.000 | 1.833 | 2.000 | 2.833 | 2.985 | 3.000 | 1.985 | 4.652 | 2.833 | 2.985 | 2.000 |
|  |  | **H_E_** | 0.530 | 0.167 | 0.409 | 0.621 | 0.621 | 0.682 | 0.303 | 0.788 | 0.591 | 0.667 | 0.409 |
|  |  | **H_O_** | 0.167 | 0.167 | 0.167 | 0.500 | 0.500 | 0.333 | 0.333 | 0.500 | 0.333 | 0.333 | 0.167 |
|  |  | **HWE** | 0.152 | 1.000 | 0.273 | 0.653 | 0.395 | 0.325 | 1.000 | 0.106 | 0.152 | 0.135 | 0.273 |
| **Merced** |  | **Alleles** | 4 | 2 | 3 | 3 | 4 | 2 | 4 | 6 | 6 | 2 | 1 |
|  |  | **A_R_** | 3.464 | 2 | 2.874 | 2.963 | 3.375 | 1.875 | 3.588 | 5.141 | 5.302 | 1.999 | 1 |
|  |  | **H_E_** | 0.598 | 0.5 | 0.607 | 0.634 | 0.527 | 0.232 | 0.723 | 0.866 | 0.857 | 0.464 | 0 |
|  |  | **H_O_** | 0.500 | 1.000 | 0.625 | 1.000 | 0.500 | 0.250 | 0.625 | 0.500 | 0.750 | 0.375 | 0 |
|  |  | **HWE** | 0.555 | 0.025 | 1.000 | 0.063 | 0.383 | 1.000 | 0.739 | 0.036 | 0.381 | 1.000 | - |
| **Mariposa** |  | **Alleles** | 3 | 3 | 8 | 6 | 4 | 3 | 4 | 6 | 9 | 5 | 2 |
|  |  | **A_R_** | 2.509 | 2.627 | 4.296 | 4.611 | 2.877 | 2.875 | 3.152 | 3.888 | 5.757 | 3.546 | 1.983 |
|  |  | **H_E_** | 0.394 | 0.563 | 0.806 | 0.769 | 0.643 | 0.631 | 0.603 | 0.729 | 0.852 | 0.665 | 0.409 |
|  |  | **H_O_** | 0.385 | 0.692 | 0.769 | 0.308 | 0.615 | 0.462 | 0.308 | 0.231 | 0.769 | 0.615 | 0.538 |
|  |  | **HWE** | 0.522 | 0.457 | 0.033 | **0.000** | 0.380 | 0.227 | 0.021 | **0.000** | 0.178 | 0.437 | 0.499 |
| **Nelder** |  | **Alleles** | 5 | 3 | 9 | 7 | 5 | 4 | 4 | 6 | 12 | 3 | 3 |
|  |  | **A_R_** | 2.598 | 2.892 | 5.205 | 3.898 | 3.510 | 3.569 | 3.535 | 3.841 | 6.110 | 2.965 | 2.925 |
|  |  | **H_E_** | 0.567 | 0.615 | 0.802 | 0.660 | 0.713 | 0.686 | 0.716 | 0.673 | 0.870 | 0.677 | 0.651 |
|  |  | **H_O_** | 0.720 | 0.680 | 0.760 | 0.280 | 0.640 | 0.240 | 0.560 | 0.480 | 0.760 | 0.440 | 0.640 |
|  |  | **HWE** | 0.189 | 0.574 | 0.548 | **0.000** | 0.438 | **0.000** | 0.001 | 0.004 | 0.169 | 0.071 | 0.194 |
| **McKinley** |  | **Alleles** | 2 | 4 | 3 | 5 | 3 | 4 | 6 | 8 | 11 | 3 | 3 |
|  |  | **A_R_** | 1.999 | 3.357 | 2.416 | 3.783 | 2.384 | 3.014 | 3.185 | 5.391 | 6.530 | 2.596 | 1.769 |
|  |  | **H_E_** | 0.508 | 0.711 | 0.562 | 0.683 | 0.557 | 0.618 | 0.462 | 0.831 | 0.889 | 0.480 | 0.151 |
|  |  | **H_O_** | 0.385 | 1.000 | 0.417 | 0.692 | 0.538 | 0.462 | 0.538 | 0.846 | 0.923 | 0.538 | 0.154 |
|  |  | **HWE** | 0.574 | 0.093 | 0.375 | 0.682 | 1.000 | 0.521 | 1.000 | 0.054 | 0.274 | 0.736 | 1.000 |
| **Cabin Creek** |  | **Alleles** | 3 | 3 | 5 | 5 | 5 | 4 | 4 | 7 | 11 | 5 | 2 |
|  |  | **A_R_** | 2.263 | 2.899 | 3.470 | 3.622 | 3.473 | 3.566 | 2.668 | 4.506 | 5.859 | 3.330 | 1.723 |
|  |  | **H_E_** | 0.539 | 0.642 | 0.698 | 0.622 | 0.708 | 0.727 | 0.465 | 0.792 | 0.855 | 0.640 | 0.193 |
|  |  | **H_O_** | 0.263 | 0.100 | 0.611 | 0.474 | 0.737 | 0.526 | 0.368 | 0.526 | 0.789 | 0.632 | 0.211 |
|  |  | **HWE** | 0.018 | 0.001 | 0.366 | 0.082 | 0.266 | 0.009 | 0.200 | 0.008 | 0.241 | 0.964 | 1.000 |
| **Converse Basin** |  | **Alleles** | 3 | 3 | 7 | 8 | 4 | 5 | 3 | 4 | 12 | 6 | 3 |
|  |  | **A_R_** | 2.907 | 2.904 | 5.054 | 5.466 | 3.200 | 3.436 | 2.554 | 3.021 | 5.873 | 4.304 | 2.113 |
|  |  | **H_E_** | 0.621 | 0.65 | 0.827 | 0.849 | 0.694 | 0.710 | 0.531 | 0.586 | 0.848 | 0.767 | 0.323 |
|  |  | **H_O_** | 0.810 | 0.524 | 0.905 | 0.571 | 0.667 | 0.762 | 0.429 | 0.429 | 0.714 | 0.714 | 0.381 |
|  |  | **HWE** | 0.347 | 0.029 | 0.132 | 0.070 | 0.363 | 0.188 | 0.602 | 0.080 | 0.038 | 0.235 | 1.000 |
| **Lockwood** |  | **Alleles** | 2 | 3 | 8 | 4 | 5 | 4 | 5 | 3 | 7 | 3 | 2 |
|  |  | **A_R_** | 1.999 | 2.997 | 6.295 | 3.838 | 4.518 | 2.963 | 4.117 | 2.624 | 4.831 | 2.929 | 1.625 |
|  |  | **H_E_** | 0.463 | 0.713 | 0.892 | 0.759 | 0.800 | 0.659 | 0.709 | 0.541 | 0.800 | 0.575 | 0.125 |
|  |  | **H_O_** | 0.625 | 0.750 | 0.875 | 0.250 | 0.750 | 0.500 | 0.625 | 0.375 | 0.750 | 0.625 | 0.125 |
|  |  | **HWE** | 0.487 | 0.841 | 0.445 | 0.011 | 0.386 | 0.379 | 0.920 | 0.217 | 0.302 | 1.000 | - |
| **Windy Gulch** |  | **Alleles** | 3 | 3 | 10 | 9 | 5 | 4 | 4 | 7 | 10 | 4 | 2 |
|  |  | **A_R_** | 2.384 | 2.877 | 5.685 | 6.536 | 4.175 | 3.658 | 3.103 | 5.565 | 5.998 | 3.007 | 1.785 |
|  |  | **H_E_** | 0.554 | 0.644 | 0.833 | 0.91 | 0.782 | 0.734 | 0.551 | 0.865 | 0.849 | 0.58 | 0.212 |
|  |  | **H_O_** | 0.615 | 0.615 | 0.769 | 0.769 | 0.615 | 0.462 | 0.308 | 0.692 | 0.846 | 0.769 | 0.231 |
|  |  | **HWE** | 0.773 | 0.380 | 0.305 | 0.210 | 0.233 | 0.015 | 0.045 | 0.420 | 0.652 | 0.274 | 1.000 |
| **Redwood Mtn** |  | **Alleles** | 3 | 3 | 6 | 8 | 5 | 3 | 4 | 6 | 8 | 5 | 1 |
|  |  | **A_R_** | 2.562 | 2.895 | 4.721 | 5.258 | 3.364 | 2.895 | 2.433 | 3.852 | 5.555 | 3.721 | 1.000 |
|  |  | **H_E_** | 0.432 | 0.607 | 0.787 | 0.822 | 0.557 | 0.645 | 0.414 | 0.702 | 0.847 | 0.715 | 0 |
|  |  | **H_O_** | 0.471 | 0.647 | 0.824 | 0.647 | 0.471 | 0.529 | 0.059 | 0.412 | 0.824 | 0.824 | 0 |
|  |  | **HWE** | 1.000 | 0.750 | 0.932 | 0.018 | 0.034 | 0.174 | 0.001 | 0.009 | 0.394 | 0.882 | - |
| **Giant Forest** |  | **Alleles** | 4 | 3 | 7 | 8 | 5 | 3 | 4 | 4 | 11 | 6 | 2 |
|  |  | **A_R_** | 2.844 | 2.794 | 5.091 | 5.258 | 4.171 | 2.914 | 2.536 | 2.841 | 6.890 | 3.856 | 1.690 |
|  |  | **H_E_** | 0.591 | 0.613 | 0.830 | 0.815 | 0.774 | 0.653 | 0.337 | 0.579 | 0.915 | 0.718 | 0.175 |
|  |  | **H_O_** | 0.625 | 0.750 | 0.733 | 0.563 | 0.750 | 0.750 | 0.250 | 0.375 | 0.800 | 0.625 | 0.063 |
|  |  | **HWE** | 1.000 | 0.656 | 0.605 | 0.029 | 0.405 | 0.006 | 0.281 | 0.019 | 0.086 | 0.407 | 0.097 |
| **Atwell Mill** |  | **Alleles** | 4 | 3 | 6 | 8 | 5 | 4 | 3 | 8 | 11 | 4 | 3 |
|  |  | **A_R_** | 3.222 | 2.257 | 3.734 | 4.812 | 4.205 | 3.302 | 2.23 | 4.834 | 6.364 | 3.562 | 2.17 |
|  |  | **H_E_** | 0.645 | 0.449 | 0.642 | 0.807 | 0.77 | 0.658 | 0.304 | 0.81 | 0.873 | 0.712 | 0.268 |
|  |  | **H_O_** | 0.500 | 0.556 | 0.667 | 0.778 | 0.944 | 0.778 | 0.111 | 0.389 | 0.889 | 0.722 | 0.278 |
|  |  | **HWE** | 0.288 | 0.142 | 0.651 | 0.866 | 0.716 | 0.560 | 0.008 | 0.001 | 0.543 | 0.163 | 1.000 |
| **Mountain Home** |  | **Alleles** | 4 | 3 | 6 | 7 | 5 | 4 | 6 | 6 | 10 | 6 | 2 |
|  |  | **A_R_** | 2.915 | 2.746 | 4.27 | 4.691 | 4.289 | 3.309 | 3.375 | 4.489 | 5.482 | 3.777 | 1.313 |
|  |  | **H_E_** | 0.537 | 0.546 | 0.741 | 0.761 | 0.768 | 0.631 | 0.506 | 0.79 | 0.822 | 0.7 | 0.063 |
|  |  | **H_O_** | 0.412 | 0.706 | 0.647 | 0.706 | 0.765 | 0.824 | 0.118 | 0.529 | 0.882 | 0.647 | 0.059 |
|  |  | **HWE** | 0.125 | 0.615 | 0.104 | 0.707 | 0.649 | 0.619 | **0.000** | 0.016 | 0.247 | 0.122 | - |
| **Black Mtn 1** |  | **Alleles** | 4 | 3 | 7 | 5 | 4 | 4 | 4 | 5 | 12 | 3 | 2 |
|  |  | **A_R_** | 3.196 | 2.861 | 4.893 | 3.581 | 3.294 | 3.046 | 3.561 | 4.431 | 7.136 | 2.877 | 1.631 |
|  |  | **H_E_** | 0.577 | 0.587 | 0.804 | 0.635 | 0.554 | 0.455 | 0.705 | 0.814 | 0.923 | 0.654 | 0.147 |
|  |  | **H_O_** | 0.692 | 0.769 | 0.769 | 0.462 | 0.462 | 0.538 | 0.385 | 0.462 | 0.846 | 0.385 | 0.154 |
|  |  | **HWE** | 1.000 | 0.602 | 0.016 | 0.099 | 0.391 | 1.000 | 0.041 | 0.003 | 0.264 | 0.014 | 1.000 |
| **Black Mtn 2** |  | **Alleles** | 4 | 3 | 6 | 6 | 4 | 3 | 4 | 6 | 12 | 5 | 3 |
|  |  | **A_R_** | 2.643 | 2.529 | 4.01 | 4.199 | 3.264 | 2.282 | 2.799 | 4.571 | 7.028 | 4.135 | 2.484 |
|  |  | **H_E_** | 0.379 | 0.548 | 0.733 | 0.754 | 0.685 | 0.433 | 0.427 | 0.767 | 0.917 | 0.743 | 0.454 |
|  |  | **H_O_** | 0.438 | 0.375 | 0.375 | 0.500 | 0.750 | 0.438 | 0.375 | 0.563 | 0.813 | 0.625 | 0.188 |
|  |  | **HWE** | 1.000 | 0.297 | 0.003 | 0.028 | 0.815 | 1.000 | 0.303 | 0.001 | 0.288 | 0.883 | 0.009 |
| **WheelMeadow** |  | **Alleles** | 3 | 4 | 7 | 10 | 4 | 6 | 4 | 10 | 13 | 4 | 2 |
|  |  | **A_R_** | 2.239 | 3.461 | 4.412 | 5.133 | 3.559 | 3.605 | 2.922 | 4.857 | 6.862 | 3.228 | 1.864 |
|  |  | **H_E_** | 0.397 | 0.706 | 0.698 | 0.798 | 0.705 | 0.642 | 0.55 | 0.779 | 0.909 | 0.68 | 0.282 |
|  |  | **H_O_** | 0.333 | 0.800 | 0.633 | 0.533 | 0.800 | 0.567 | 0.600 | 0.300 | 0.900 | 0.767 | 0.333 |
|  |  | **HWE** | 0.332 | 0.649 | 0.572 | 0.001 | 0.900 | 0.157 | 0.793 | **0.000** | 0.479 | 0.949 | 0.563 |
| **Packsaddle** |  | **Alleles** | 4 | 2 | 5 | 5 | 3 | 3 | 4 | 3 | 7 | 3 | 1 |
|  |  | **A_R_** | 3.263 | 2 | 3.747 | 3.878 | 2.893 | 1.957 | 3.000 | 2.500 | 5.268 | 2.878 | 1 |
|  |  | **H_E_** | 0.658 | 0.526 | 0.656 | 0.695 | 0.637 | 0.336 | 0.621 | 0.563 | 0.837 | 0.584 | 0 |
|  |  | **H_O_** | 0.400 | 0.600 | 0.700 | 0.400 | 0.400 | 0.400 | 0.800 | 0.400 | 0.700 | 0.400 | 0 |
|  |  | **HWE** | 0.078 | 1.000 | 0.103 | 0.017 | 0.208 | 1.000 | 0.354 | 0.307 | 0.092 | 0.242 | - |
| **Grant** |  | **Alleles** | 4 | 2 | 4 | 1 | 2 | 2 | 2 | 3 | 2 | 2 | 2 |
|  |  | **A_R_** | 2 | 2 | 4 | 1 | 2 | 2 | 2 | 3 | 2 | 2 | 2 |
|  |  | **H_E_** | 0.5 | 0.5 | 1 | 0 | 0.5 | 0.5 | 0.5 | 1 | 0.5 | 0.5 | 0.5 |
|  |  | **H_O_** | 2 | 2 | 2 | 0 | 1 | 1 | 1 | 1 | 1 | 0 | 1 |
|  |  | **HWE** | - | - | - | - | - | - | - | - | - | - | - |
| **South Fork** |  | **Alleles** | 4 | 3 | 5 | 3 | 4 | 4 | 4 | 6 | 6 | 5 | 2 |
|  |  | **A_R_** | 3.652 | 2.985 | 4.636 | 2.985 | 3.833 | 4.000 | 3.970 | 5.333 | 6.000 | 4.652 | 1.985 |
|  |  | **H_E_** | 0.561 | 0.621 | 0.742 | 0.621 | 0.773 | 0.648 | 0.773 | 0.803 | 0.867 | 0.788 | 0.303 |
|  |  | **H_O_** | 0.500 | 0.833 | 0.833 | 0.500 | 0.833 | 0.600 | 0.667 | 0.500 | 1.000 | 0.833 | 0.333 |
|  |  | **HWE** | 0.516 | 0.636 | 0.538 | 0.394 | 1.000 | 0.048 | 0.469 | 0.065 | 0.019 | 0.140 | 1.000 |
| **Deer Creek** |  | **Alleles** | 4 | 3 | 2 | 2 | 2 | 2 | 3 | 3 | 4 | 5 | 1 |
|  |  | **A_R_** | 4 | 3 | 2 | 2 | 2 | 2 | 3 | 3 | 4 | 5 | 1 |
|  |  | **H_E_** | 0.75 | 0.6 | 0.4 | 0.2 | 0.5 | 0.55 | 0.675 | 0.4 | 0.825 | 0.825 | 0 |
|  |  | **H_O_** | 0.600 | 0.600 | 0 | 0.200 | 0.200 | 0.400 | 0.400 | 0.200 | 0.400 | 0.600 | 0 |
|  |  | **HWE** | 1.000 | 0.619 | 0.111 | - | 0.333 | 1.000 | 0.365 | 0.116 | 0.187 | 0.466 | - |

Alleles: Allele number; A_R_: Allelic richness; H_E_: expected heterozygosity; H_O_: observed heterozygosity; HWE: Hardy-Weinberg expectations; bold/shaded cells indicate a significant deviation from Hardy-Weinberg expectations.

Table S2 Linkage disequilibrium among alleles. Probabilities shown in bold significant after Bonferroni correction

|  | Placer | N Calaver | S Calaver | Tuol | Merc | Mari | Neld | McKin | Cab Crk | Conv Bas | Lock | Wind Gul | Red Mtn | Giant For | Atwell Mill | Mtn Home | Black Mtn1 | Black Mtn2 | Wheel Meadw | Pack | South Fork | Deer Crk |
| --- | --- | --- | --- | --- | --- | --- | --- | --- | --- | --- | --- | --- | --- | --- | --- | --- | --- | --- | --- | --- | --- | --- |
| 1-2 | - | 0.981 | 0.246 | 0.134 | 0.275 | - | 0.592 | 0.765 | 0.804 | 0.879 | 0.225 | 0.684 | 0.367 | 0.2635 | 0.754 | 0.553 | 0.569 | 0.773 | 0.148 | 0.995 | 0.353 | 0.121 |
| 1-3 | - | 0.001 | 0.111 | 0.566 | **0.0001** | 0.182 | 0.326 | 0.256 | 0.244 | 0.0001 | 0.944 | 0.394 | 0.001 | 0.318 | 0.809 | 0.083 | 0.009 | 0.434 | 0.412 | 0.541 | 0.190 | 0.008 |
| 1-4 | - | 0.770 | 0.071 | 0.012 | **0.0001** | 0.985 | 0.038 | 0.371 | 0.365 | 0.007 | 0.423 | 0.186 | 0.129 | 0.005 | 0.064 | 0.095 | 0.072 | 0.108 | 0.285 | 0.009 | 0.210 | 0.833 |
| 1-5 | - | 0.366 | 0.349 | 0.042 | 0.008 | 0.435 | 0.202 | 0.995 | 0.385 | 0.405 | 0.785 | 0.307 | 0.269 | 0.161 | 0.130 | 0.157 | 0.184 | 0.429 | 0.305 | 0.633 | 0.028 | 0.013 |
| 1-6 | - | 0.001 | 0.203 | 0.001 | 0.171 | 0.039 | 0.262 | 0.002 | 0.0001 | 0.026 | 0.701 | 0.558 | 0.210 | 0.339 | 0.922 | 0.060 | 0.012 | 0.431 | 0.753 | 0.505 | 0.009 | 0.006 |
| 1-7 | - | 0.152 | 0.097 | 0.002 | 0.001 | 0.221 | 0.004 | 0.410 | 0.324 | 0.111 | 0.164 | 0.4902 | 0.851 | 0.008 | 0.986 | 0.286 | 0.0001 | 0.664 | 0.258 | 0.101 | 0.001 | 0.186 |
| 1-8 | - | 0.638 | 0.026 | 0.001 | **0.0001** | 0.019 | 0.139 | 0.349 | 0.142 | 0.215 | 0.404 | 0.505 | 0.017 | 0.083 | 0.512 | 0.070 | 0.001 | 0.415 | 0.554 | 0.401 | 0.189 | 0.011 |
| 1-9 | - | 0.004 | 0.137 | 0.302 | 0.347 | 0.172 | 0.226 | 0.003 | 0.059 | 0.003 | 0.949 | 0.694 | 0.708 | 0.407 | 0.236 | 0.044 | 0.642 | 0.540 | 0.278 | 0.191 | 0.111 | 0.011 |
| 1-10 | - | 0.143 | 0.354 | 0.002 | **0.0001** | 0.088 | 0.914 | 0.627 | 0.482 | 0.004 | 0.827 | 0.618 | 0.920 | 0.862 | 0.974 | 0.138 | 0.122 | 0.008 | 0.438 | 0.870 | 0.005 | 0.003 |
| 1-11 | - | 0.735 | 0.411 | 0.085 | - | - | 0.177 | 0.914 | 0.401 | 0.060 | 0.900 | 0.500 | 0.847 | - | 0.412 | 0.280 | 0.703 | 0.814 | 0.213 | - | 0.824 | - |
| 2-3 | - | 0.173 | 0.308 | 0.306 | 0.226 | - | 0.956 | 0.132 | 0.559 | 0.985 | 0.177 | 0.264 | 0.298 | 0.235 | 0.204 | 0.206 | 0.657 | 0.897 | 0.015 | 0.726 | 0.735 | 0.074 |
| 2-4 | 0.445 | 0.101 | 0.333 | 0.017 | 0.037 | - | 0.034 | 0.287 | 0.838 | 0.182 | 0.047 | 0.016 | 0.187 | 0.292 | 0.396 | 0.026 | 0.511 | 0.536 | 0.040 | 0.041 | 0.388 | 0.648 |
| 2-5 | 0.443 | 0.632 | 0.243 | 0.233 | 0.053 | - | 0.181 | 0.559 | 0.814 | 0.892 | 0.063 | 0.033 | 0.034 | 0.118 | 0.659 | 0.565 | 0.546 | 0.587 | 0.229 | 0.638 | 0.595 | 0.001 |
| 2-6 | - | 0.230 | 0.735 | 0.208 | 0.380 | - | 0.530 | 0.257 | 0.979 | 0.329 | 0.127 | 0.556 | 0.110 | 0.166 | 0.865 | 0.577 | 0.886 | 0.391 | 0.502 | 0.085 | 0.804 | 0.219 |
| 2-7 | - | 0.009 | 0.004 | 0.428 | 0.433 | - | 0.524 | 0.169 | 0.950 | 0.903 | 0.239 | 0.583 | 0.249 | 0.062 | 0.244 | 0.322 | 0.008 | 0.707 | 0.791 | 0.801 | 0.187 | 0.003 |
| 2-8 | 0.781 | 0.834 | 0.233 | 0.051 | 0.766 | - | 0.505 | 0.003 | 0.778 | 0.849 | 0.371 | 0.180 | 0.226 | 0.315 | 0.585 | 0.732 | 0.902 | 0.072 | 0.223 | 0.045 | 0.452 | 0.001 |
| 2-9 | 0.558 | 0.141 | 0.031 | 0.013 | 0.095 | - | 0.510 | 0.166 | 0.219 | 0.858 | 0.203 | 0.227 | 0.360 | 0.225 | 0.303 | 0.539 | 0.329 | 0.547 | 0.096 | 0.637 | 0.731 | 0.121 |
| 2-10 | 0.317 | 0.619 | 0.274 | 0.675 | 0.247 | - | 0.862 | 0.004 | 0.524 | 0.870 | 0.390 | 0.092 | 0.961 | 0.014 | 0.086 | 0.614 | 0.954 | 0.061 | 0.006 | 0.295 | 0.567 | 0.030 |
| 2-11 | - | 0.632 | 0.205 | 0.306 | - | - | 0.392 | 0.490 | 0.821 | 0.889 | 0.062 | 0.522 | 0.485 | - | 0.100 | 0.930 | 0.695 | 0.833 | 0.152 | 0.584 | 0.831 | - |
| 3-4 | - | 0.453 | 0.074 | 0.408 | **0.0001** | 0.991 | 0.0001 | **0.0001** | 0.015 | 0.210 | 0.162 | 0.0001 | 0.144 | 0.038 | 0.033 | 0.732 | 0.002 | 0.080 | 0.105 | 0.046 | 0.203 | 0.352 |
| 3-5 | - | 0.214 | 0.192 | 0.085 | 0.773 | 0.539 | 0.778 | 0.004 | 0.080 | 0.394 | 0.636 | 0.006 | 0.297 | 0.966 | 0.009 | 0.283 | 0.002 | 0.008 | 0.286 | 0.127 | 0.567 | 0.067 |
| 3-6 | - | **0.0001** | 0.0003 | 0.726 | 0.498 | 0.650 | 0.020 | **0.0001** | 0.440 | 0.100 | 0.219 | 0.117 | 0.058 | 0.329 | 0.086 | 0.824 | 0.036 | 0.309 | 0.085 | 0.813 | 0.085 | 0.023 |
| 3-7 | - | **0.0001** | 0.761 | 1 | 0.084 | 0.605 | 0.011 | 0.013 | 0.003 | 0.366 | 0.239 | 0.045 | 0.397 | 0.061 | 0.119 | 0.675 | 0.0001 | 0.0001 | 0.0002 | 0.552 | 0.183 | 0.020 |
| 3-8 | - | 0.626 | 0.056 | 0.017 | 0.020 | 0.251 | 0.002 | 0.677 | 0.002 | 0.045 | 0.361 | 0.093 | 0.133 | 0.135 | 0.511 | 0.205 | 0.002 | 0.023 | 0.003 | 0.016 | 0.051 | 0.377 |
| 3-9 | - | **0.0001** | 0.189 | 0.016 | 0.596 | 0.427 | 0.001 | 0.032 | 0.001 | 0.217 | 0.004 | 0.087 | 0.150 | 0.280 | 0.012 | 0.257 | 0.077 | 0.001 | 0.003 | 0.095 | 0.001 | 0.001 |
| 3-10 | - | **0.0001** | 0.556 | 0.278 | **0.0001** | 0.608 | 0.016 | 0.0001 | 0.055 | 0.002 | 0.520 | 0.307 | 0.810 | 0.268 | 0.151 | 0.325 | 0.001 | 0.001 | 0.009 | 0.568 | 0.020 | **0.0001** |
| 3-11 | - | 0.503 | 0.144 | 0.514 | - | - | 0.597 | 0.235 | 0.002 | 0.888 | 0.488 | 0.132 | 0.632 | - | 0.002 | 0.764 | 0.738 | 0.293 | 0.002 | 0.286 | 0.364 | - |
| 4-5 | 0.138 | 0.763 | 0.022 | 0.083 | 0.090 | 0.820 | 0.919 | 0.050 | 0.018 | 0.538 | 0.824 | **0.0001** | 0.001 | **0.0001** | 0.034 | 0.423 | 0.138 | 0.029 | 0.035 | 0.168 | 0.014 | 0.416 |
| 4-6 | - | 0.040 | 0.006 | 0.234 | 0.374 | 0.905 | 0.013 | 0.001 | 0.498 | 0.028 | 0.504 | 0.019 | 0.033 | 0.017 | 0.230 | 0.440 | 0.150 | 0.315 | 0.017 | 0.0001 | 0.323 | 0.704 |
| 4-7 | - | 0.021 | 0.506 | 0.194 | 0.008 | 0.787 | 0.016 | 0.001 | 0.042 | 0.028 | 0.013 | 0.001 | 0.002 | **0.0001** | 0.141 | 0.142 | **0.0001** | 0.107 | 0.056 | 0.724 | 0.053 | 0.074 |
| 4-8 | 0.007 | 0.317 | **0.0001** | 0.041 | 0.001 | 0.746 | 0.003 | 0.066 | 0.074 | 0.001 | **0.0001** | **0.0001** | 0.0002 | 0.0003 | 0.007 | 0.001 | 0.006 | 0.046 | 0.307 | 0.008 | 0.054 | 0.648 |
| 4-9 | 0.004 | 0.809 | 0.446 | 0.046 | 0.112 | 0.531 | 0.006 | **0.0001** | 0.331 | 0.001 | 0.499 | 0.0001 | 0.056 | **0.0001** | 0.024 | 0.127 | 0.378 | 0.279 | 0.007 | 0.006 | 0.110 | 0.003 |
| 4-10 | 0.186 | 0.434 | 0.436 | 0.033 | **0.0001** | 0.538 | 0.199 | **0.0001** | 0.265 | 0.087 | 0.001 | 0.025 | 0.030 | 0.083 | 0.008 | 0.263 | 0.001 | 0.116 | 0.073 | 0.267 | 0.158 | 0.810 |
| 4-11 | - | 0.344 | 0.937 | 0.189 | - | - | 0.470 | 0.001 | 0.448 | 0.413 | 0.390 | 0.106 | 0.021 | - | 0.137 | 0.105 | 0.908 | 0.933 | 0.165 | 0.296 | 0.831 | - |
| 5-6 | - | 0.214 | 0.314 | 0.142 | 0.964 | 0.594 | 0.248 | 0.127 | 0.745 | 0.088 | 0.827 | 0.322 | 0.042 | 0.168 | 0.488 | 0.689 | 0.101 | 0.166 | 0.900 | 0.387 | 0.020 | 0.081 |
| 5-7 | - | 0.351 | 0.670 | 0.477 | 0.003 | 0.010 | 0.004 | 0.136 | 0.141 | 0.460 | 0.783 | 0.272 | 0.067 | 0.066 | 0.269 | 0.001 | 0.013 | 0.102 | 0.431 | 0.870 | 0.006 | 0.001 |
| 5-8 | 0.373 | 0.084 | **0.0001** | 0.079 | 0.008 | 0.003 | 0.012 | 0.153 | 0.587 | 0.103 | 0.240 | 0.007 | 0.001 | 0.001 | 0.137 | 0.005 | 0.049 | 0.047 | 0.085 | 0.051 | 0.049 | **0.0001** |
| 5-9 | 0.443 | 0.578 | 0.164 | 0.087 | 0.001 | 0.027 | 0.181 | 0.029 | 0.262 | 0.307 | 0.349 | 0.016 | 0.009 | 0.222 | 0.120 | 0.084 | 0.304 | 0.022 | 0.060 | 0.334 | 0.563 | 0.030 |
| 5-10 | 0.329 | 0.854 | 0.515 | 0.247 | 0.377 | 0.005 | 0.323 | 0.001 | 0.424 | 0.125 | 0.153 | 0.172 | 0.637 | 0.145 | 0.409 | 0.028 | 0.056 | 0.072 | 0.282 | 0.286 | 0.058 | 0.001 |
| 5-11 | - | 0.401 | 0.273 | 0.305 | 0.401 | - | 0.170 | 0.099 | 0.277 | 0.045 | 0.454 | 0.326 | 0.269 | - | 0.022 | 0.869 | 0.180 | 0.442 | 0.356 | 0.956 | 0.273 | - |
| 6-7 | - | 0.003 | 0.160 | 0.009 | 0.712 | 0.407 | 0.0004 | **0.0001** | 0.244 | 0.033 | 0.280 | 0.265 | 0.001 | 0.016 | 0.027 | 0.094 | 0.002 | 0.123 | 0.696 | 0.913 | 0.002 | 0.251 |
| 6-8 | - | 0.003 | 0.018 | 0.001 | 0.238 | 0.077 | **0.0001** | 0.007 | 0.116 | 0.0001 | 0.728 | 0.434 | 0.160 | 0.001 | 0.727 | 0.165 | 0.185 | 0.540 | 0.102 | 0.097 | 0.001 | 0.074 |
| 6-9 | - | **0.0001** | 0.007 | 0.015 | 0.188 | 0.223 | 0.131 | 0.001 | 0.039 | 0.180 | 0.166 | 0.100 | 0.102 | 0.018 | 0.073 | 0.839 | 0.459 | 0.172 | 0.075 | 0.417 | 0.163 | 0.004 |
| 6-10 | - | 0.003 | 0.135 | 0.010 | 0.096 | 0.323 | 0.723 | 0.095 | 0.755 | 0.096 | 0.120 | 0.370 | 0.300 | 0.036 | 0.186 | 0.179 | 0.389 | 0.275 | 0.532 | 0.886 | 0.038 | 0.009 |
| 6-11 | - | 0.214 | 0.486 | 0.147 | - | - | 0.388 | 0.328 | 0.832 | 0.647 | 0.451 | 0.248 | 0.970 | - | 0.003 | 0.804 | 0.854 | 0.316 | 0.491 | 0.872 | 0.768 | - |
| 7-8 | - | 0.485 | 0.083 | 0.107 | 0.012 | 0.046 | **0.0001** | **0.0001** | 0.065 | 0.086 | 0.013 | 0.517 | 0.010 | 0.001 | **0.0001** | **0.0001** | 0.057 | 0.001 | 0.156 | 0.060 | 0.005 | 0.002 |
| 7-9 | - | 0.022 | 0.009 | 0.540 | 0.301 | 0.147 | 0.125 | 0.001 | 0.050 | 0.014 | 0.717 | 0.155 | 0.097 | 0.226 | 0.014 | 0.015 | **0.0001** | **0.0001** | 0.013 | 0.017 | 0.349 | 0.003 |
| 7-10 | - | 0.001 | 0.050 | 0.013 | 0.007 | 0.086 | 0.353 | 0.006 | 0.134 | 0.029 | 0.119 | 0.119 | 0.001 | 0.388 | 0.001 | 0.374 | **0.0001** | 0.002 | 0.380 | 0.730 | 0.001 | 0.004 |
| 7-11 | - | 0.703 | 0.413 | 0.129 | - | - | 0.596 | 0.315 | 0.022 | 0.415 | 0.042 | 0.045 | 0.226 | - | 0.628 | 0.293 | 0.957 | 0.477 | 0.019 | 0.037 | 0.670 | - |
| 8-9 | 0.584 | 0.925 | 0.233 | 0.001 | 0.058 | 0.051 | **0.0001** | **0.0001** | **0.0001** | 0.220 | 0.073 | 0.004 | 0.030 | 0.031 | 0.001 | 0.004 | 0.848 | 0.002 | 0.025 | 0.308 | 0.052 | 0.069 |
| 8-10 | 0.199 | 0.005 | 0.157 | 0.002 | 0.001 | 0.171 | 0.001 | 0.061 | 0.010 | 0.411 | 0.669 | 0.700 | 0.062 | 0.247 | 0.001 | 0.071 | 0.003 | 0.001 | 0.006 | 0.001 | 0.279 | 0.020 |
| 8-11 | - | 0.939 | 0.536 | 0.062 | - | - | 0.299 | 0.402 | 0.020 | 0.861 | 0.587 | 0.434 | 0.531 | - | 0.045 | 0.034 | 0.34 | 0.080 | **0.0001** | 0.457 | 0.528 | - |
| 9-10 | 0.148 | 0.001 | 0.058 | 0.040 | 0.002 | 0.002 | 0.357 | 0.001 | 0.062 | 0.035 | 0.012 | 0.251 | 0.797 | 0.859 | 0.001 | 0.001 | 0.724 | **0.0001** | 0.402 | 0.528 | 0.007 | 0.002 |
| 9-11 | - | 0.376 | 0.501 | 0.207 | - | - | 0.411 | 0.068 | **0.0001** | 0.466 | 0.954 | 0.591 | 0.533 | - | **0.0001** | 0.344 | 0.819 | 0.175 | 0.001 | 0.943 | 0.391 | - |
| 10-11 | - | 0.746 | 0.328 | **0.0001** | - | - | 0.794 | 0.001 | 0.060 | 0.317 | 0.117 | 0.360 | 0.250 | - | 0.921 | 0.155 | 0.799 | 0.297 | **0.0001** | 0.1790 | 0.459 | - |

Table S3. Assignments to clusters of numbers of individuals from the different groves of origin according to BAPS spatial analysis

| Cluster | 1 | 2 | 3 | 4 | 5 | 6 | 7 | 8 | 9 | 10 | 11 |
| --- | --- | --- | --- | --- | --- | --- | --- | --- | --- | --- | --- |
| Placer | 8 |  |  |  |  |  |  |  |  |  |  |
| N Calaveras |  |  | 1 |  |  |  |  |  | 15 |  |  |
| S Calaveras |  |  |  |  | 20 |  |  |  |  |  |  |
| Tuolomne |  |  |  | 5 |  |  |  |  |  |  | 1 |
| Merced |  | 8 |  |  |  |  |  |  |  |  |  |
| Mariposa |  |  |  |  |  |  |  |  |  | 11 | 2 |
| Nelder |  |  | 1 | 3 |  |  | 21 |  |  |  |  |
| McKinley |  |  | 11 |  |  |  | 1 |  |  |  |  |
| Cabin Creek |  |  | 1 |  |  |  |  |  |  |  | 18 |
| Converse Basin |  |  |  | 1 |  |  |  |  |  |  | 20 |
| Lockwood |  |  |  |  |  |  |  |  |  |  | 8 |
| Windy Gulch |  |  |  |  |  |  |  |  |  |  | 13 |
| Grant |  |  |  |  |  |  |  |  |  |  | 2 |
| Redwood Mtn |  |  |  |  |  |  |  |  |  |  | 17 |
| Giant Forest |  |  |  |  |  |  |  |  |  |  | 16 |
| Atwell Mill |  |  |  |  |  |  |  |  |  |  | 18 |
| Mountain Home |  |  |  |  |  |  |  | 2 |  |  | 6 |
| Black Mtn 1 |  |  |  |  |  | 1 |  |  |  |  | 16 |
| Black Mtn 2 |  |  |  |  |  |  |  |  |  |  | 30 |
| Wheel Meadow |  |  |  |  |  |  |  |  |  |  | 13 |
| Packsaddle |  |  |  |  |  |  |  |  |  |  | 14 |
| South Fork |  |  |  |  |  |  |  |  |  |  | 10 |
| Deer Creek |  | 1 |  |  |  | 1 |  |  |  |  | 3 |

Table S4. Priors for all demographic parameters used in DIYABC

| Parameters | Prior range | Conditions |
| --- | --- | --- |
| N1 | Uniform (10-1000) |  |
| N2 | Uniform (10-1000) | N2<N1 |
| N3 | Uniform (10-1000) | N3<N1 |
| N4 | Uniform (10-1000) | N4<N1 |
| N5 | Uniform (10-1000) | N5<N1 |
| tcal | Uniform (10-100) |  |
| db | Uniform (1-100) |  |
| 5b | Uniform (1-100) |  |
| tmer | Uniform (10-100) | tmer>tcal |
| 4b | Uniform (1-100) |  |
| tnel | Uniform (10-100) | tnel>tmer |
| 3b | Uniform (1-100) |  |
| t1 | Uniform (10-1000) | t1>tnel |
| 2b | Uniform (1-100) |  |
| tanc | Uniform (10-1000) | tanc>td |
| Nanc | Uniform (10-100000) | Nanc>Ndiv |
| tdiv_1_ | Uniform (10-1000) |  |
| Ndiv | Uniform (10-1000) |  |
| tdiv_2_ | Uniform (10-10000) | t2>td |
| Ndiv2 | Uniform (10-10000) | Ndiv2>Ndiv |
| Nanc2 | Uniform (10-100000) | Nanc2>Nanc, Nanc2>Ndiv2 |


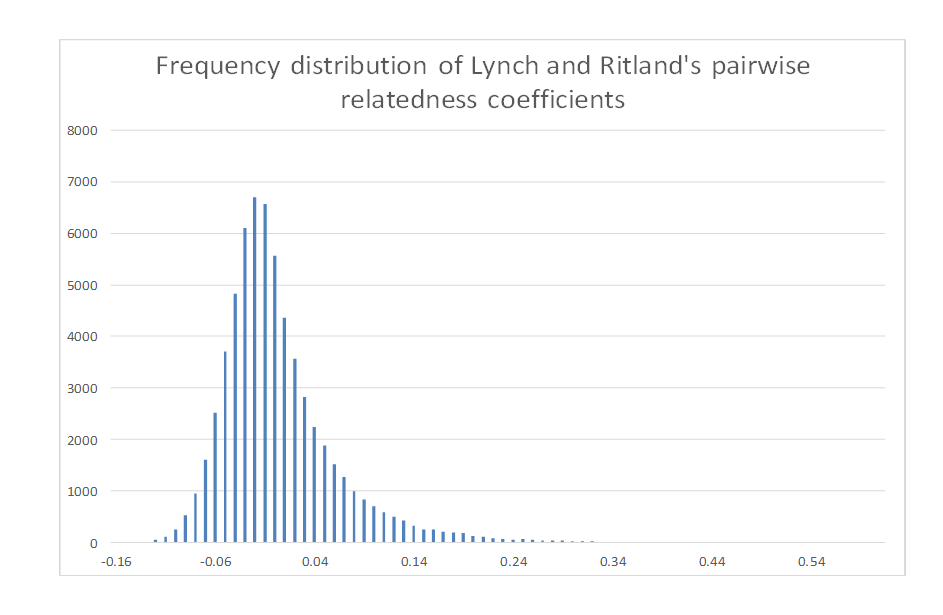


Fig S1. Frequency distribution of pairwise relatedness coefficients


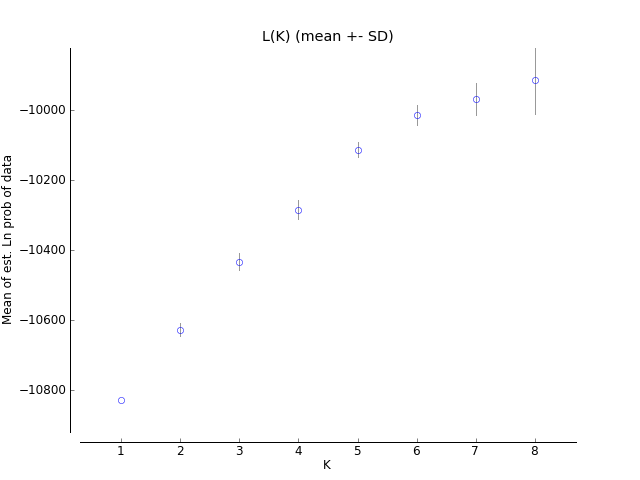


Fig S2. Log probability of the data for increasing values of K in STRUCTURE


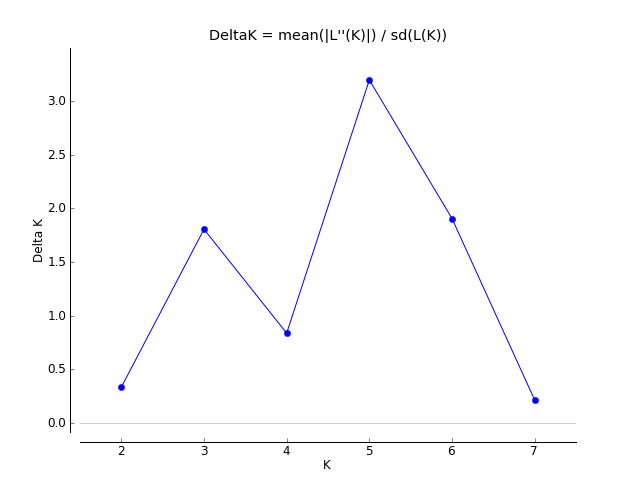


Fig S3. Evanno *et al.* (2005) ΔK parameter for increasing values of K


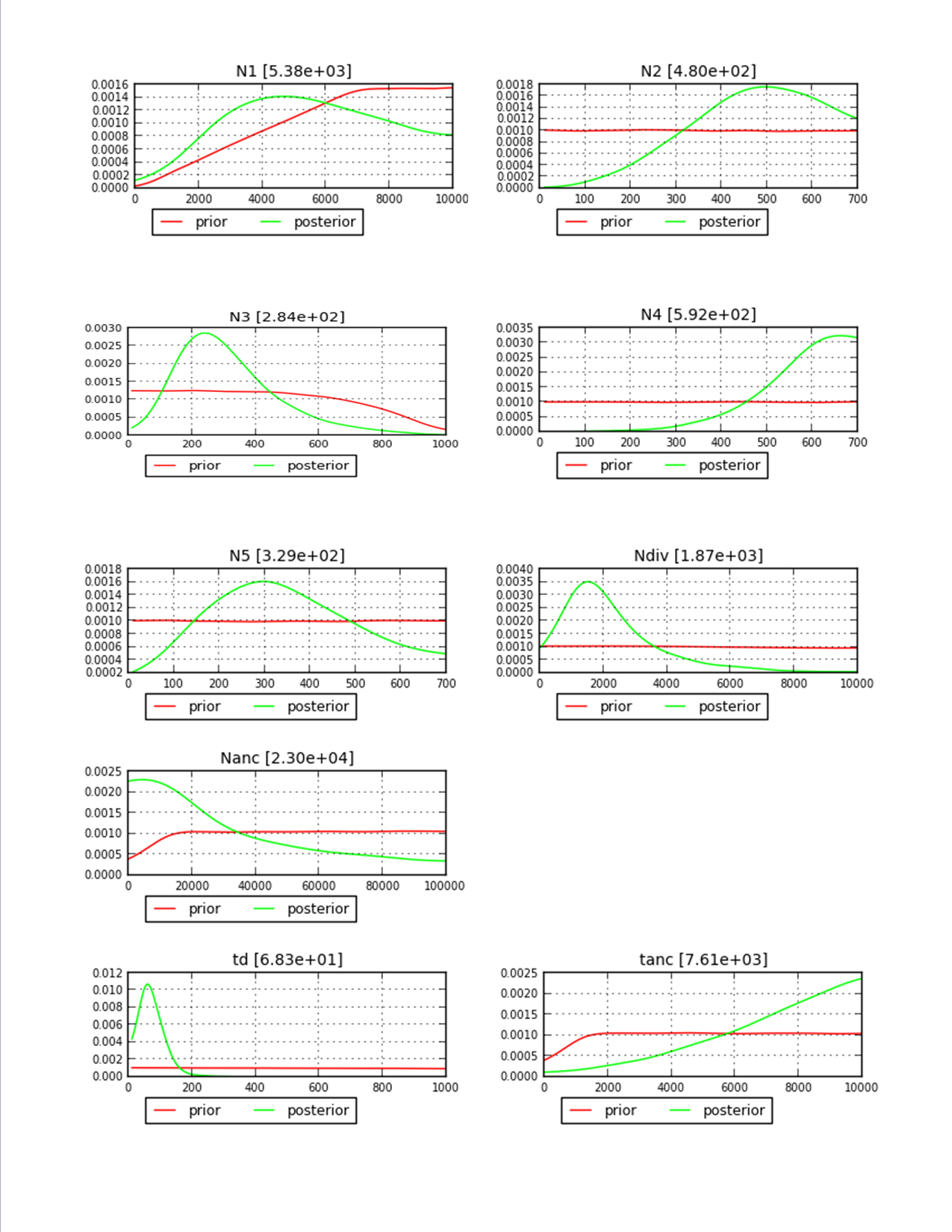


Fig S4 Parameter estimates using DIYABC
